# Chronological T‐wave alternation before and after the onset of arrhythmogenic right ventricular cardiomyopathy

**DOI:** 10.1111/anec.12965

**Published:** 2022-06-02

**Authors:** Akira Sato, Hirofumi Saiki, Maki Kudo, Yurie Takizawa, Seiko Kuwata, Satoshi Nakano, Yumi Sato, Kunihiko Miura, Kotaro Oyama, Manami Akasaka

**Affiliations:** ^1^ Department of Pediatrics Iwate Medical University Shiwa Japan; ^2^ Department of Clinical Laboratory Iwate Health Service Association Morioka Japan; ^3^ Department of Pediatrics Iwate prefectural Miyako Hospital Miyako Japan; ^4^ Michinoku Medical Center on Disability and Health Shiwa Japan

**Keywords:** arrhthmogenic right ventricular cardiomyopathy, heart failure, one and one‐half repair, school cardiac screening, sudden cardiac death, T wave

## Abstract

Identification of arrhythmogenic right ventricular cardiomyopathy (ARVC) during childhood is challenging due to the lack of specific ECG manifestation. We report chronological ECG alteration before several years of the ARVC onset in two affected children. Their ECG at the age of 6 years was almost normal for their age, and their chronological ECGs exhibited inversion of T wave in inferior leads, which are typical for ARVC, developed at younger age than that in precordial leads. In addition, the leftmost T‐wave inversion in the precordial lead shifted toward the left in our patients, which is a sharp contrast to its physiological transition.

## Graphical abstract

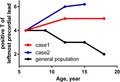

## 1 INTRODUCTION

Arrhythmogenic right ventricular cardiomyopathy (ARVC) is a myocardial disease characterized by progressive myocardial degradation and fibrofatty replacement, where genetic background affects its pathophysiology (Ganatra & Sharma, 2017). Since ARVC is closely associated with sudden cardiac death in young individuals and athletes, its early diagnosis, and implementation of timely intervention should be considered to prevent cardiovascular events. While accumulating evidence emphasized the importance of characteristic ECG waveform in the diagnosis of ARVC in adults, diagnosis before presentation of typical ECG waveform or myocardial impairment is challenging. Although T‐wave inversion in the precordial leads is among the established diagnostic criteria, there is a paucity of information on when and how to assess T‐wave alternation during childhood (Marcus et al., 2010). Here, we report two cases of ARVC whose ECG waveforms could be followed‐up before and after their diagnosis of ARVC. Written informed consents for use of medical record as well as school cardiac screening record were obtained from both patients and guardians.

## 2 CASE PRESENTATION

### 2.1 Patient 1

**FIGURE 1:**
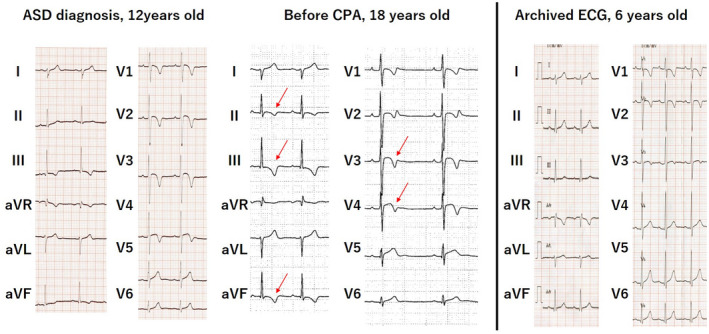
ECG in patient 1. T waves in leads III and V1‐3 were negative at 6 years old (c), then those in leads III, aVf and V1‐4 became negative and that in lead II was attenuated at 12 years old (a) when ASD was diagnosed. While inverted T wave in inferior leads became further pronounced at the age of 18 (b), those in precordial leads were, inversely, attenuated, which might be due to suppressed right ventricular volume load

The patient was referred to our hospital after being diagnosed with atrial septal defect (ASD) at the age of 12 years. He had an unremarkable history, and his diagnosis was confirmed by ECG findings of right axis deviation coupled with right ventricular hypertrophy at the school cardiac screening (Figure 1a), and subsequent echocardiogram. T‐wave inversion in leads III, aVf and V1‐V4 was considered as the result of right ventricular enlargement due to ASD at this point (Izumida et al., 2000). When he underwent percutaneous ASD closure at the age of 16 years, inversion of T wave in inferior leads could be clearly recognized (Figure 1b). Although premature ventricular contractions were observed early after procedure, it became in remission in a few days, thus he was followed‐up as an outpatient. The echocardiogram showed mild right ventricular dilatation with excellent left ventricular contraction of the ejection fraction at more than 65%. While he stated neither chest pain nor palpitation, he had cardiopulmonary arrest during exercise at the age of 18 years. He was successfully resuscitated after four sessions of automated electric defibrator (AED), then recovered after a few days of percutaneous cardiopulmonary support without neurological complication. Since recorded ECG waveform in AED device exhibited ventricular fibrillation and his myocardial biopsy was compatible with ARVC, intracardiac defibrillator was implanted.

The archived ECG at the initial school cardiac screening at 6 years of age (Figure 1c) revealed normal T‐wave polarity as his age, although T wave at the diagnosis of ASD was inverted in leads V1‐V4 and inferior leads. Interestingly, the subsequent follow‐up revealed progression of T‐wave inversion in inferior leads, whereas that in precordial leads appeared to have slightly regressed at the age of 18 years.

### 2.2 Patient 2

**FIGURE 2:**
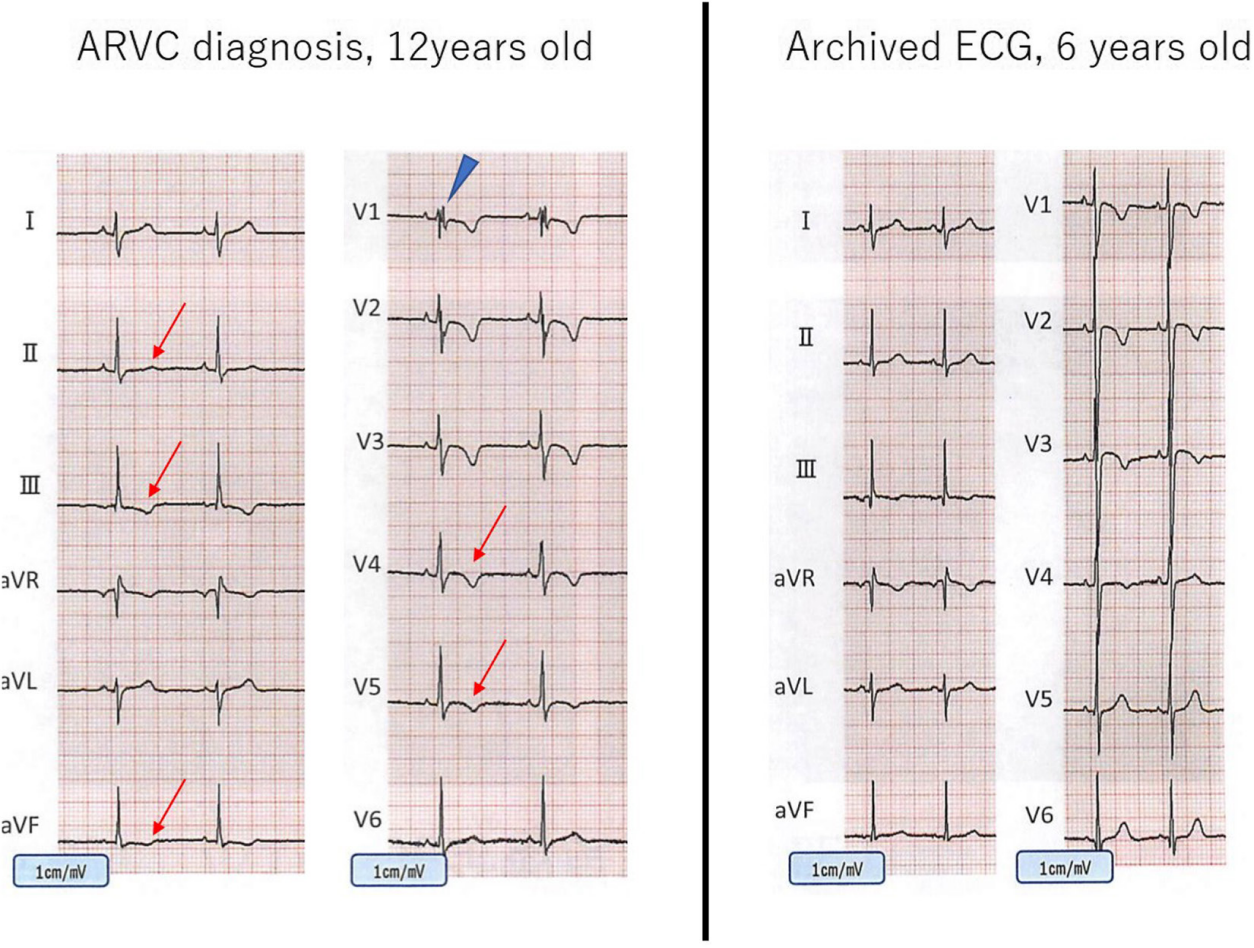
ECG in patient 2. T wave in leads III and V1‐3 was negative at 6 years old (b), but subsequent assessment at 12 years old revealed that those in leads III, aVf, and V1‐4 became negative (a) when marked RV dilatation with suppressed contractility was identified. At this point, LV contractility was preserved and late potentials were positive

A 13‐year‐old boy was referred to our hospital due to T‐wave abnormality at school cardiac screening. He had a history of NICU admission due to neonatal infection, but otherwise he was healthy without any symptom of heart failure. At the first visit to the hospital, his ECG exhibited normal sinus rhythm of 67 bpm without cardiac axis deviation (Figure 2a). The QRS wave exhibited RSR pattern with inverted T wave in leads V1‐V4 and flattened in lead V5. The chest X‐ray showed mild cardiomegaly with the cardiothoracic ratio of 54%, and echocardiogram indicated marked dilatation of right ventricle with suppressed contractility. No finding of pulmonary hypertension was observed. A Holter ECG confirmed polymorphic premature ventricular contractions, which convinced us of the diagnosis of ARVC (Marcus et al., 2010). Since intensive medical interview implied chest discomfort and plasma concentration of brain type natriuretic peptide was as high as 141 pg/mL, we prescribed exercise restriction as well as oral anti‐heart failure medications including carvedilol and diuretics. Cardiac MRI indicated marked suppression of right ventricular ejection fraction of 4%, with delayed Gadolinium enhancement in the interventricular septum. Although he was tolerable to the Master double exercise test, polymorphic PVCs were induced, thus we strictly limited exercise at this point. Despite exercise restriction and anti‐heart failure medications, his chest discomfort got worse. Hence, bidirectional cardiopulmonary shunt was performed to suppress right ventricular eccentric remodeling.

Although archived ECG of the school cardiac screening at 6 years of age, which was taken approximately 6 years before diagnosis, showed inverted T wave in lead III, it was regarded as non‐specific at that point (Figure 2b). In sharp contrast, T‐wave inversion disseminated to left precordial (V4, V5) and inferior (II,III, aVf) leads, and iRBBB pattern was newly recognized at the diagnosis of ARVC (Figure 2a).

## 3 DISCUSSION

ARVC is characterized by the fibrofatty replacement of myocardium due to genetic background, thus the affected patients may develop part of their symptoms during childhood (Marcus et al., 1982). Roudigik et al. reported increasing number of pediatric patients with ARVC but emphasized its diagnostic difficulty (Roudijk et al., 2020). Since exercise is known to exacerbate myocardial derangement in patients with ARVC, early identification of affected patients would be of importance to prevent progression of myocardial degradation. While repolarization and depolarization abnormalities are parts of primary components of the current Task Force criteria for ARVC diagnosis, those can only be applied for individuals aged more than 14 years, which is a significant barrier for the early diagnosis in pediatric patients (Marcus et al., 2010; Roudijk et al., 2020). By taking advantage of school cardiac screening system in Japan, archived ECGs taken years before the onset of ARVC could be retrieved. In the review of two patients, we found that the typical T‐wave morphology in the precordial leads was not observed at several years before onset. Interestingly, T‐wave inversion of inferior leads antecedent to that in precordial leads.

**FIGURE 3:**
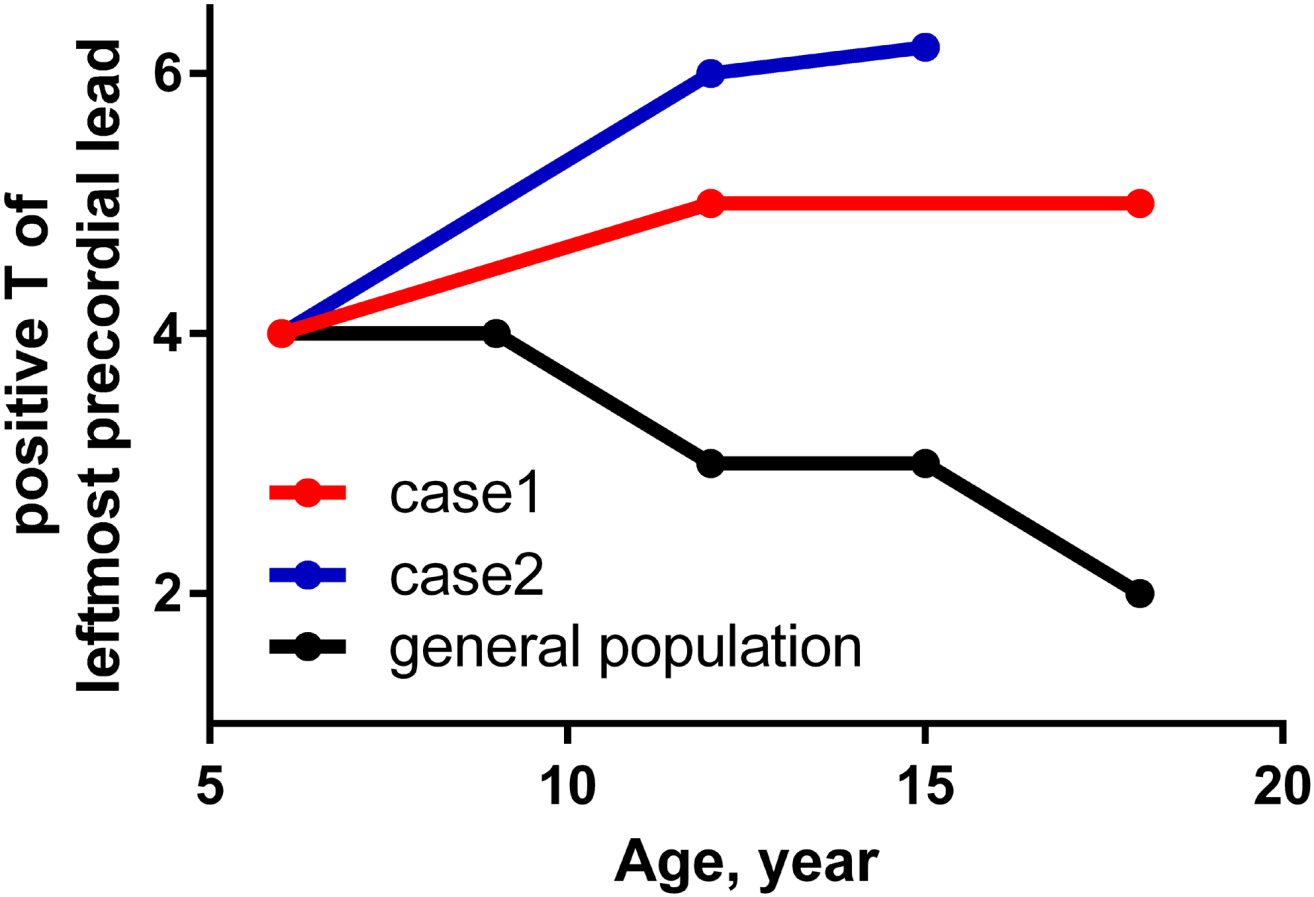
The trend of positive conversion of the T wave in the precordial leads. The age in the horizontal axis and corresponding leftmost lead with positive T wave in the vertical axis were shown. While the physiological positive conversion of T wave, where more than 90% of general cohort show positive T wave, progresses to the right precordial leads in the normal cohort, those in the affected cohort regress with aging

Repolarization abnormality represented by the inversion of T wave in right precordial leads is listed as major and minor criteria in those older than 14 years (Marcus et al., 2010). Since inverted T waves in the right precordial leads in children are often normal due to their age or physical development (Anselmi et al., 2021), it is difficult to distinguish physiological variant from manifestation of myocardial diseases including ARVC (Saguner et al., 2015). Our two cases, for the first time, clearly indicated that the distribution of T‐wave inversion in precordial leads was normal initially, which markedly changed to the typical waveform for ARVC after several years of follow‐up, highlighting the limited role of T wave in precordial leads in the early screening of ARVC. As shown in the first patient, diagnosis could become further challenging when complications predispose to right ventricular volume load (Izumida et al., 2000). In addition, his T‐wave polarity in leads V1‐4 appeared to have regressed slightly after closure of ASD. Indeed, repolarization abnormalities may disappear during follow‐up in patients with isolated ARVC (Saguner et al., 2015). Assuming such limitations, chronological changes of polarity in the precordial leads might help in the diagnosis of ARVC as the distribution of T‐wave inversion could regress against normal cardiac development (Figure 3).

Meanwhile, Saguner et al. emphasized that the T‐wave inversion in the inferior leads might be further specific for the diagnosis of ARVC (Saguner et al., 2015). Consistent with and further strengthening their study, T wave in inferior leads were already inverted even before years of developing ARVC in our two cases, although this might cause over‐triage if the diagnosis is only based on this finding. At least, periodical follow‐up of these children would be warranted as the inversion of T wave in inferior‐lateral leads is uncommon even in pediatric athletes (D'Ascenzi et al., 2019).

## 4 CONCLUSIONS

The ECGs at 6 years of age were within normal range in patients developing ARVC. Our cases highlighted that chronological ECG follow‐up, particularly in inferior leads, would contribute to early identification of affected patients. The prospective research would be warranted to determine the usefulness of chronological ECG follow‐up particularly in the inferior leads in the early identification of patients with ARVC.

## CONFLICTS OF INTEREST

The authors declare no conflicts of interest.

## AUTHOR CONTRIBUTION

AS and HS contributed to conceptualization, data collection and drafted manuscript. MK, YT, SK, SN and KM collected the latest and archived data, and analyzed with constructive discussion. KO supervised patients' management and conceptualization. YS and MA contributed to constructive discussion. All the authors helped drafting manuscript and approved it.

## ETHICAL APPROVAL

Written informed consent was obtained from both patients and guardians.

## Data Availability

The data that support the findings of this study are available from [Iwate Medical University]. Restrictions apply to the availability of these data, which were used under license for this study. Data are available [from the authors] with the permission of [Iwate Medical University].
